# Chlamydia trachomatis testing among young people: what is the role of stigma?

**DOI:** 10.1186/s12889-015-2020-y

**Published:** 2015-07-14

**Authors:** Kevin A. T. M Theunissen, Arjan E. R. Bos, Christian J. P. A. Hoebe, Gerjo Kok, Stan Vluggen, Rik Crutzen, Nicole H. T. M. Dukers-Muijrers

**Affiliations:** Department of Sexual Health, Infectious Diseases and Environmental Health, Public Health Services South Limburg, Geleen, The Netherlands; Faculty of Psychology and Educational Sciences, Open University, Heerlen, The Netherlands; Department of Medical Microbiology Maastricht Infection Centre (MINC), School for Public Health and Primary Care (CAPHRI), Maastricht University Medical Centre (MUMC+), Maastricht, The Netherlands; Department of Work & Social Psychology, Maastricht University, P.O. Box 616 6200 MD, Maastricht, The Netherlands; Department of Health Promotion, School for Public Health and Primary Care (CAPHRI), Maastricht University, Maastricht, The Netherlands

**Keywords:** Chlamydia, Stigma, Stigma management, Testing, Disclosure, Encouragement

## Abstract

**Background:**

To reach young people for *Chlamydia trachomatis* (CT) testing, new web-based strategies are used to offer testing via young people’s sexual and social networks. The success of such peer-driven strategies depends on whether individuals disclose their own testing and encourage others to get tested. We assessed whether public- and self-stigma would hamper these behaviours, by comparing anticipations and experiences relating to these issues in young men and women who already tested or never tested for CT.

**Methods:**

Participants were recruited at an STI clinic and two schools in the Netherlands. Semi-structured interviews were analysed from 23 sexually active heterosexual young people between 16–24 years using qualitative content analysis with a framework approach.

**Results:**

Both tested and never tested participants perceived public stigma and anticipated shame and self-stigma in relation to testing. Maintaining good health was identified as main reason for testing. Never tested and tested participants anticipated that they would feel shame and receive stigmatizing reactions from people outside their trusted network if they would disclose their testing, or encourage them to test. From a selected group of trusted peers, they anticipated social support and empathy. When tested participants disclosed their testing to trusted peers they did not experience stigma. Due to the fact that no one disclosed their testing behaviour to peers outside their trusted network, stigma was avoided and therefore tested participants reported no negative reactions. Similarly, regarding the encouragement of others to test, most tested participants did not experience negative reactions from sex partners and friends.

**Conclusions:**

Young people perceive public stigma and anticipate self-stigma and shame in relation to CT testing, disclosure and encouraging others to test. People do test for CT, including those who anticipate stigma. To avoid stigmatizing reactions, stigma management strategies are applied, such as selective disclosure and the selective encouragement of others to test (i.e. only in a small trusted peer network). Care strategies that deploy sexual and social networks of individuals can reach into small networks surrounding a person. These strategies could be improved by exploring methods to reach high-risk network members outside the small trusted circle of a person.

## Background

*Chlamydia trachomatis* (CT) is a much underestimated sexually transmitted infection (STI) and worldwide diagnoses of CT have increased in recent years [1–4]. CT is the most diagnosed bacterial STI among sexually active people, and has potential reproductive sequelae. A major risk group for CT are young heterosexual people below 25 years of age. They are targeted by health care professionals using key control strategies that encompass testing, treating, and partner notification (PN), to interrupt the inherent transmission chain [5, 6]. Several associated factors, which influence the uptake of these strategies, have been identified. A systematic review on CT testing in young women revealed that the uptake of CT testing was impeded by ignorance and inaccurate information, denial, moral connotations, stigma, fear, anxiety, confidentiality and privacy concerns, and pragmatic factors such as cost and test discomfort [7]. Other barriers among women and men include the embarrassment and shame associated with seeking care [8, 9], the asymptomatic nature of the infection [6, 10], fear of a positive test result [6, 11], and perceived STI-related stigma (i.e., fear of being subjected to negative societal attitudes and discrimination) [12]. Facilitators included accurate knowledge, feelings of personal relevance, multiple test options (i.e., home-based test kits, self-administered swabs), free tests, and support with diagnosis [7]. The process of PN is also influenced by several factors. Barriers to notifying a sex partner, as revealed in a systematic review of PN, included stigma, guilt, blame, possible relationship breakdown, violence, and missing contact details [13]. Partner notification can be facilitated by using Patient-Delivered Partner Therapy (i.e., partners are treated via the patients by providing prescriptions or medications without a medical personal evaluation), online notification, and home-based test kits [13].

To overcome barriers and explore the facilitators of CT testing and PN among young people, innovative care methods have been developed in addition to the regular care provided by STI clinics and general practitioners in for instance Europe Australia, and US [13–20]. These new methods include web-based programmes that utilise email and text messaging, alongside home-based sampling [18–20]. These programmes aim to facilitate testing in high-risk groups of individuals who are connected in sexual networks and/or social networks. Peers from such networks surrounding CT positive individuals are important targets in CT control, as they typically show similar high risk, for example with respect to unprotected sex or sex with a CT positive person [21]. Communication in social networks is associated with the sexual behaviour among friends in these networks [22]. Modelling may also play a role; the actual behaviour of peers may influence a person’s behaviour, for example regarding sexual behaviour, but also regarding CT testing [23]. Therefore, essential components of care methods that aim to reach high-risk networks include the disclosure of CT testing behaviour to sexual and social network members and the encouragement to get them tested using for example the internet and home-based sampling. Successful disclosure and encouragement could thus potentially lead to better partner notification and an increase in CT testing and emotional support among peers [24, 25].

Web-based CT care programs focus on utilising the factors identified as facilitators for CT testing and PN. However, factors that have been identified as barriers, especially stigma [15, 25–28], may still be of concern in the processes of disclosure and encouragement. A stigma is a deeply discrediting attribute that results in widespread social disapproval [29]. It consists of two fundamental components -- the recognition of difference, and devaluation [30]. Stigmatization has different manifestations that can be overt (e.g., social rejection, avoidance) as well as subtle (e.g., lack of eye contact) [29]. *Public stigma* refers to the cognitive, affective and behavioural responses of other people towards an individual who possesses a stigmatized characteristic. Public stigma can lead to *self-stigma* which refers to an individual’s awareness of his/her stigmatized condition, the social devaluation connected with his/her condition, and the possible internalization of this stigma [29]. Public stigma may shape anticipated stigma in persons with a stigmatized condition. However, anticipated stigma does not automatically translate into the actual experience of stigma.

Previous studies about stigma surrounding the disclosure of test behaviour have focused on anticipations or experiences with stigma by young men or women surrounding STI in general [25], HIV [31] and the Human Papilloma Virus [26]. Previous research on stigma surrounding CT was conducted in a UK population with a wide age-range [32], limiting the generalizability of the results to young populations. The present study assessed both anticipated stigma and experiences with stigma in young (16–24 years old) heterosexual men and women who had either never been tested (from here on referred to as “never tested”) or who had been tested (referred to as “tested”) for CT. By assessing the role of stigma in CT testing, disclosure of testing behaviour and encouragement of others to get tested, it is hoped that results will inform new care methods that aim to increase CT testing in high-risk groups, and thereby improve CT control.

## Methods

### Design and setting

A qualitative study was conducted using semi-structured interviews by researchers of the Public Health Service South Limburg and Maastricht University, the Netherlands. Participants provided written informed consent. According to national guidelines a Research Ethics Board reviewed and approved this study and gave permission to interview 16 and 17 year olds without parental consent (Ethics Committee Psychology Maastricht University, reference number 13-4-054). This article adheres to the RATS guidelines on qualitative research [33].

### Recruitment of participants

Sexually active heterosexual people between 16 and 25 years were eligible for participation. Between May and August 2013, both never CT tested (n = 13) and CT tested (n = 10) young people were recruited for participation. Tested young people were recruited during an STI clinic visit by nurses performing their sexual health consultations. Never tested young people were recruited during a sexual health education lesson at a secondary school and via leaflets at a University, both in the same region as the area served by the STI clinic.

### Data collection

Semi-structured interviews lasting approximately 30 min, were conducted over the telephone and tape-recorded. Data were collected using a semi-structured interview protocol, which was constructed in line with the opinion of experts in the field of social psychology and stigma and a review of the literature. All questions were related to CT and addressed topics related to the anticipations and experiences surrounding testing, disclosure, and the encouragement of peers to test (Table 1). Saturation occurred at around 20 interviews, and later interviews served to confirm themes identified earlier in the analysis.

**Table 1:** Subject and topic list about Chlamydia testing, disclosure of own testing behaviour and encouragement of others to test, among tested and never tested young people

Subject	Topic
General	Talking about sex and sexually transmitted infections with others
CT testing	Experiences of others with testing
	Opinion about others who have tested
	Opinion about others who are CT positive
	Thoughts about testing
	Feelings about testing
	Reason(s) for testing
	Feelings about test results
Disclosure	Experiences of others regarding the disclosure of testing and results
	Disclosure of testing to others
	Disclosure of test results to others
	Reasons to disclose testing to others
	Reasons to disclose test results to others
	Reactions of others after disclosure
Encouragement	Encouragement of sex partners to test
	Encouragement of friends to test
	Reactions of sex partner and friends

### Analyses

The audio-recorded interviews were transcribed verbatim in Dutch and analysed independently by two interviewers (SV and KT) using the "framework" approach [34], which involves structured stages of data management, descriptive accounts and explanatory accounts. Several transcripts were explored in detail, in order for SV and KT to become familiar with the data, after which open coding was applied using NVivo software. Codes were then applied to subsequent transcripts and grouped into categories: CT testing, disclosure of CT testing, and encouragement of peers to get tested. Furthermore, associations within categories were sought. Any disagreements found in the analysis were resolved through discussion, and consensus was reached by PS and KT consulting a third party (i.e. ND and AB).

## Results

### Sample characteristics

The sample consisted of 25 interviewed participants of whom two had to be excluded, because they were not eligible for participation. Of the remaining participants (n = 23), all were sexually active and heterosexual, 13 were female, 22 were of Dutch nationality, and the overall mean age was 20 years old (age range 16–24 years). Of the 10 participants who had ever been tested, 7 had tested positive for CT. Participants who had been tested had undergone between 1 and 5 CT tests before participating in the current study.

### Anticipated stigma surrounding own testing

Almost all *tested* and *never tested* participants perceived public stigma. In several cases this was based on what they had observed or what they heard had happened to other young people, i.e. those who had received stigmatized reactions after they went for STI testing or were found to be STI positive.

Interviewee: “Well, yes, I think that people do have certain ideas if someone has an STI, or get certain ideas about that person”.

Interviewer: “OK, and what kind of ideas would they be?”

Interviewee: “That there are prejudices attached, so that you are maybe seen as easy, or that sort of thing… That you are more likely to have sex with others, and that you don’t think about it so much.”

*(Tested, female no 2, 18 years).*

“That people respond to it (CT infection) differently, react strangely, at school a girl had it and she was always being called names … that girl was always called a slut or things like that.”

*(Never tested, female no 22, 16 years)*

The perception of public stigma among *never tested* participants was in line with the reported anticipated feelings of shame when going for a CT test, but differed from the good experiences with actual testing in *tested* participants. Most *never tested* participants thought testing would indicate unsafe sex or would be associated with a positive test result. Others were afraid of the testing procedure and the possibility of a positive test result. Despite these factors, most *never tested* participants said that they would test if they have had unsafe sex or experienced symptoms, because they valued their own health and that of their peers. Furthermore, some also mentioned certainty as a reason to be tested, which was also stated among most *tested* participants. Even though *tested* participants were nervous in the beginning, and did not know what to expect, both CT positives and CT negatives stated that they were glad to know their CT status.

Interviewee: “If I had to do a test like that then I would probably feel a bit ashamed, because then you, yes, you’ve maybe caught something, and then you’re a bit ashamed”

Interviewer: “And what exactly would you be ashamed of?”

Interviewee: “Yeah, that you just didn’t pay attention really and that you haven’t been safe”

*(Never tested, female no 9, 20 years).*

“Um, if I had unsafe sex for some reason or another, I would like to know if I’d got anything, so for yourself it’s sensible, and also for your health, to know if you have anything – and also for the other, should you have had sex”

*(Never tested, male no 14, 21 years).*

*Never tested* participants reported that they expected to feel bad if they would receive a positive test result. However, they would also be relieved, because at that moment they could act upon the test result and find appropriate care. Some *tested* participants who were CT positive expressed feelings of shame or anger towards their sex partners. *Never tested *participants would feel relieved if they received a negative test result, which was comparable with the feelings of most of the *tested* participants who indeed turned out to test CT negative. Similar responses were observed between male and female participants regarding stigma and own CT testing

“Well, I was really ashamed… that I had caught something like that and hadn’t taken better care”

*(Tested, female no 18, 21 years)*

### Disclosure of CT testing

All participants who had *never tested* reported that they would disclose CT testing and test results only to parents, good friends and sex partners whom they trusted, and from whom they expected a positive reaction (social support and empathy). Likewise, all participants who had *tested* stated that they had only disclosed their test and its result to their trusted peers (parents, friends, sex partners), receiving only positive reactions. *Tested* participants reported that it was self-evident to them that they inform sex partners. *Tested* participants received positive reactions from their trusted peers (parents, friends, sex partners). *Never tested* and *tested* participants would not disclose to a broader network, because they anticipated shame and stigmatizing reactions, including gossiping and insulting language. For the *never* tested participants shame was associated with CT positivity and risky sexual behaviour. Due to the fact that no one disclosed their testing behaviour to peers outside the trusted network, experienced stigma from these people was avoided and *tested* participants reported no negative reactions. Similar responses were observed between men and women regarding their disclosure of test behaviour and results.

Interviewer: “And do you expect a reaction from them (good friends) when you tell them (about the test and results)?”

Interviewee: “Yes you could call it empathy what you expect, but also that they can help you or that they have gone through something similar that makes it easier for you to cope, as it were.”

*(Never tested, male no 17, 23 years)*

“Yeah, I’m not someone who would just throw that out there. That’s what I have my most trusted friend for, one or two. And with them I talk about it and then I ask advice and then for me it’s fine, actually.”

*(Tested, male no 23, 20 years).*

“It is something about myself, not everyone needs to know that I have it… Yeah, I won’t be called a little slut, or have people think that I jump into bed with everyone or something.”

*(Tested, female no 21, 22 years)*

### Encouraging others to get tested

Most of the *never tested* participants indicated that, if they happened to be CT positive, they would encourage sex partners to test, because they want to prevent transmission or would be concerned about their own and their partner’s health. Likewise, all *tested* participants had already, or would in the future personally encourage sex partners to test although they would find it difficult to do so. Some *tested* participants had themselves been encouraged by their sex partner or by someone within their trusted network to test for CT. A few tested participants and peers visited the clinic together for testing.

Interviewer: “And why do you find it important that she is also tested?”

Interviewee: “Yeah, if I have it then she can also have it and imagine that she has it and then we do it again, and I’ll have it again.”

*(Never tested, male no 5, 17 years)*

“The last time that I had myself tested was because I heard via a former sex partner that he had got Chlamydia and that I should also be tested.”

*(Tested, female no 1, 21 years)*

Despite anticipated negative reactions such as anger or shock, *nevertested* participants thought that their sex partners would go for a CT test once they had been encouraged by the participant to do so. This is in line with the positive experiences among *tested* participants where several sex partners they had talked to also got CT tested. *Tested* participants had not experienced negative reactions when encouraging others to test.

“I think that they (sex partners) would also be a bit angry, yeah, I would be too, like yeah, how can it have happened? But on the other hand I also really think that it’s good that I tell him so that he can get himself tested.”

*(Never tested, female no 9, 20 years)*

Most of the *never tested* and *tested* participants anticipated that they would also encourage a trusted network of good friends to test; friends with whom they already talk about sex or STIs or who they know have had unsafe sex or multiple sex partners. Some *nevertested *participants anticipated negative reactions like laughing or anger if they would encourage a friend to get tested. Several *nevertested* participants expressed the opinion that it was the responsibility of these friends to test for CT when necessary. Most *tested* participants did not anticipate negative reactions from friends and some had already encouraged friends to test. Similar responses were observed between men and women regarding the encouragement of others to test for CT.

“Yeah, you could of course start talking about it, but if you haven’t, for example, ever talked about sex or something like that with a person, that person’s not a good friend, but a stranger or someone, then you’re not going to suddenly say you should have a Chlamydia test done, anyway, that’s not something I would do.”

*(Never tested, male no 11, 17 years)*

“Yes, I expect that they would maybe also be slightly angry that I have the idea that they maybe have Chlamydia, but I think that they would probably take that on board and let themselves be tested after all, just to be sure.”

*(Never tested, female no 22, 16 years)*

“I know in this case that my female friends… that it is the case that if someone said to me, well yes, I went to Spain last month on holiday and I had unsafe sex there, that I could just say, well yeah, I would go to the GGD [*STI clinic]*, if I were you. In that sense our group is very straightforward about things like that and we are not ashamed about it.”

*(Tested, female no 25, 24 years)*

## Discussion

The present study explored the role of stigma in relation to CT testing, the disclosure of testing to peers, and the encouragement of peers to get tested, in a group of young never tested and tested heterosexual individuals.

Perceived public stigma and anticipated self-stigma were reported regardless of whether people had ever tested for CT or not. Nevertheless, all participants either intended to test when needed or had indeed tested already. This finding is in line with some other studies among tested and never tested young people in clinics, community based organizations, and household samples, where shame was not found to be a barrier to STI testing [12, 35]. A study among young women in general practitioners settings and family planning clinics demonstrated that fear of stigmatization if they accepted screening did not lead to rejection of CT testing when it was offered to them [36].

Tested and never tested participants disclosed both their test behaviour and, when tested, their test result to a small, trusted network of peers. They anticipated positive reactions such as support and empathy from this small network. Similar results have been found in a study among young Irish adults who informed a few “key individuals” after their testing experience, because they helped them to feel normal and gave them emotional support [25]. Among the tested participants in this study, it was self-evident that they disclosed their CT testing and results to their sex partners, which is in line with previous studies about PN and STIs [13]. Despite the potential self-stigma and public stigma associated with notifying sex partners, many young people consider partner notification in practice “the right thing to do”, and people’s experiences with PN were much better than they had initially expected [13, 27, 37]. Stigmatizing reactions and shame were anticipated among all participants when disclosing details of their CT testing and results to a “non-trusted” broader network of peers. However, it has been shown that people try to minimize or regulate the negative psychological and social impact of stigma by using problem-focused and emotion-focused coping strategies [38, 39]. In the present study, participants used selective disclosure as such stigma management strategy, and indeed no tested participants in this study had experienced stigmatizing reactions. Fear of stigmatization and the selective disclosure to a few key individuals has also been found in other studies regarding the disclosure of STI testing and test results [25, 26], and moreover in studies investigating stigma associated with mental illnesses [40].

Disclosure does not automatically lead to the encouragement of peers to test for CT. Novel approaches that deploy social and sexual networks in order to get high-risk individuals tested for CT place importance on pro-active encouragement among young people [17]. Never tested participants reported that they would encourage sex partners to get tested if they themselves tested CT positive. This finding is in line with the experience of tested participants who were either encouraged by peers or who themselves had encouraged friends or sex partners to get tested. Again, stigma management strategies resulted in the encouragement only of a select trusted circle of sex partners and friends to test for CT.

In contrast to findings in other studies [32, 41], in our study, men did not report less CT-related stigma or shame compared to women. No differences were observed between men and women regarding their stigma management strategies.

### Recommendations

People do test for CT, including those who anticipate stigma. Despite anticipated stigma social and sexual networks can be reached for testing. Therefore, care strategies that deploy sexual and social networks to reach high-risk young people with CT testing are potentially effective. Yet, their effectiveness will potentially be limited by the small size of trusted networks reached due to people’s stigma management strategies. With selective disclosure and encouragement (to get tested) young people protect themselves from anticipated stigma from peers outside their trusted peer networks. Nonetheless, a person outside an individual’s trusted network may still be reached by such care strategies, because he or she may be a trusted peer in someone else’s network. Care methods, such as web-based applications that already reach high risk trusted networks, will benefit from including ways to overcome anticipated stigma without increasing the impact of experienced stigma from outside these trusted networks. For example, via anonymous disclosure and encouragement, or by the use of home-based sampling kits, which have been shown to greatly facilitate the management of sexual partners [15, 42]. Sexual health care providers such as nurses and physicians have also an important role to play in the motivation and guidance of young people disclosing to their peers and encouraging them to get tested [43]. Care providers are generally trained in Motivational Interviewing (MI). MI targets the intrinsic motivation of people to change behaviour by supporting people to examine and resolve ambivalence to their needs and abilities [44]. MI is already used in PN, yet its use can be explored to reach trusted social networks but even more, to reach non-trusted networks.

### Strengths and weaknesses of the present study

One of the strengths of this study is the inclusion of encouragement of peers. Several studies assessing stigma have been conducted regarding the disclosure of CT testing to sex partners and friends, but to our knowledge none of these have included encouragement of peers. A further strength of this study is the comparison between the hypothetical behaviour and anticipations among never tested young people with the real behaviour and experiences of tested young people. One possible limitation of our study is that all our tested participants were from Dutch STI clinics and all our never tested participants were from the general community (i.e., secondary school and university), and these groups might differ regarding STI knowledge and/or sexual risk behaviour. Nevertheless, we found that perceived public stigma and behavioural intentions regarding testing, disclosing and encouragement were largely similar between our tested and never tested participants. By including tested and untested young people from different backgrounds and gender, we do consider it likely that the results of this study provide a general theoretical understanding of how stigma plays a role in testing and disclosure that may be exported to provide explanatory theory for the experiences of other individuals who are in comparable situations. It is unknown whether results can be extrapolated to other target groups (i.e. men having sex with men or commercial sex workers) and to other STIs (i.e. syphilis and HIV), because of differences in sexual risk behaviour and severity of the illness, and possible related differences in anticipated stigma. Therefore, caution is warranted with regard to the generalization of these findings to other target groups than young heterosexuals and other STI than CT.

## Conclusion

Young people perceive public stigma, they anticipate self-stigma and feelings of shame when they test for CT, disclose their test behaviour to peers, and encourage their peers to get tested. Nevertheless, despite these potential barriers, young people have been tested, or have expressed the intention to test for, CT. As a protection against anticipated stigmatizing reactions, people use stigma management strategies such as selective disclosure and the encouragement of only a small trusted peer network to test for CT. Care strategies that deploy the sexual and social networks of individuals can reach into small networks surrounding a person. These strategies could be improved by exploring methods to reach high-risk network members outside the small trusted circle of a young person.

### Key messages

- Young people perceive public stigma with Chlamydia testing
- Despite feelings of shame and anticipated self-stigma, young people would be willing to test for Chlamydia
- Stigma surrounding disclosure of testing behaviour and encouragement of others to test is anticipated. However the experience of stigma is effectively avoided by only disclosing to a small trusted peer group.
- Young people use stigma management strategies such as selective disclosure and selective encouragement of trusted others to protect themselves from stigmatizing reactions
- Care strategies that deploy social and sexual networks to invite young people to test for Chlamydia need to take into account the small size of these trusted peer networks.

## Abbreviations

CT: Chlamydia trachomatis
STI: Sexual Transmitted Infection
PN: Partner Notification
HIV: Human immunodeficiency virus
